# Changes in the relative thickness of individual subcutaneous adipose tissue layers in growing pigs

**DOI:** 10.1186/1751-0147-49-32

**Published:** 2007-11-07

**Authors:** Fintan J McEvoy, Anders B Strathe, Mads T Madsen, Eiliv Svalastoga

**Affiliations:** 1 Department of Small Animal Clinical Sciences, Faculty of Life Sciences, University of Copenhagen, 32 Dyrlaegevej, Frederiksberg C, DK-1870, Denmark; 2 Department of Basic Animal and Veterinary Sciences, Faculty of Life Sciences, University of Copenhagen, 7 Groennegaardsvej, Frederiksberg C, DK-1870, Denmark; 3 Danish Pig Production, 3 Axeltorv, Copenhagen, DK-1609, Denmark

## Abstract

**Background:**

The thickness of the subcutaneous fat layer is an important parameter at all stages of pig production. It is used to inform decisions on dietary requirements to optimize growth, in gilts to promote longevity and finally to assist in the calculation of payments to producers that allow for general adiposity. Currently for reasons of tradition and ease, total adipose thickness measurements are made at one or multiple sites although it has been long recognized that up to three well defined layers (outer (L1), middle (L2), and inner (L3)) may be present to make up the total. Various features and properties of these layers have been described. This paper examines the contribution of each layer to total adipose thickness at three time points and describes the change in thickness of each layer per unit change in body weight in normal growing pigs.

**Methods:**

A group of nine pigs was examined using 14 MHz linear array transducer on three separate occasions. The average weight was 51, 94 and 124 kg for each successive scan. The time between scanning was approximately 4 weeks. The proportion of each layer to total thickness was modeled statistically with scan session as a variable and the change in absolute thickness of each layer per unit change in body weight was modeled in a random regression model.

**Results:**

There was a significant change in ratios between scans for the middle and inner layers (*P *< 0.001). The significant changes were seen between the first and second, and between the first and final, scan sessions. The change in thickness per unit change in body weight was greatest for L2, followed by L1 and L3.

**Conclusion:**

These results demonstrate that subcutaneous adipose layers grow at different rates relative to each other and to change in body weight and indicate that ultrasound can be used to track these differences.

## Background

Measurements of subcutaneous adipose tissue are used in decision making during pig production for optimal growth, for longevity in gilts and for quality control and carcass classification post mortem [1-4]. Typically these measurements are made using ultrasound. Transducer frequencies of 3.5 to 7 MHz are reported for this application with data displayed as an image for B-mode (brightness mode) and as a number or numbers indicating either the total adipose thickness or the thickness of individual layers for A-mode (amplitude mode). The scan site and the use of a depth measurement that includes all fat layers are historically based as these sites and parameters were measured either by palpation or by sharp dissection prior to the advent of the use of ultrasound.

Adipose tissue deposited at the scan site used in this paper is present in either two or three layers depending on the condition of the animal. Some work has previously been published describing the biochemical differences between these layers [5]. More recently their genetic relationship and the predictability value of these individual layers, for commercially interesting traits have been reported [6]. Detecting the thickness of individual layers non-invasively is of interest as it may allow the practice of examining individual layers as opposed to total thickness, to be adopted as part of routine management. We wished to determine if all layers grow at the same rate or if changes in the relative thicknesses of the layers occur to such an extend that they are reliably detected using ultrasound. A knowledge of the relative growth of these layers in normal production pigs should lead to a better understanding of their role and potential for monitoring body composition. While ultrasound permits evaluation of individual layers this is not normal practice. Instead a single measurement is made of skin plus total subcutaneous adipose thickness ("back fat thickness").

Accurate image based recognition of individual adipose layers relies on there being clear and sharp margins between adjacent layers and is enhanced by any difference in appearance there may be between layers. Ultrasound image quality is in part a function of transducer frequency but also of transducer and machine design [7]. Diagnostic ultrasound units providing state of the art image quality are not suited to use in commercial farming but do provide optimal image quality of subcutaneous adipose deposits.

This paper describes the use of optimal quality ultrasound imaging to track the occurrence and relative growth of the individual adipose tissue layers in growing pigs.

## Methods

### Animals

Nine female landrace – large white crossbred pigs (all from different litters) were each ultrasonographically scanned on three occasions (scan number 1, 2 and 3) approximately 4 weeks apart. Scans were performed as part of a larger study involving a computer tomography scan and the taking of blood samples and biopsies. Sedation was thus required and achieved using Azaperone (Stresnil, Mallinckrodt, USA), 0.1 to 0.2 mg/kg i.m. Pigs were fed a normal production diet (114 FEsv/100 Kg) *ad lib *and ranged in weight from 49 Kg at the first scan to 140 Kg at the final session. Mean body weight (standard deviation in parenthesis) for the group was 51.4 (2.4), 93.8 (7.2) and 124.1 (11.2) Kg for the first, middle and final scans respectively. During scanning the pigs were maintained in sternal recumbency. The study was approved by the Danish National Animal Ethics Council.

### Ultrasonography

An Acuson Sequoia (Siemens, Germany) ultrasound unit fitted with a 7 to 14 MHz linear array transducer was used. The machine was set to the default "small parts" setting and transducer frequency to 14 MHz. The scanning site was at the level of the last rib on the right side, 7 cm from the midline (referred to as the P2 site). Prior to scanning the hair was clipped and skin defatted with alcohol. Acoustic coupling gel was then applied directly to the prepared skin. Images were saved in DICOM format for later analysis.

### Data handling and image analyses

DICOM images were imported into an open source image analysis program ImageJ [8]. The image analysis measuring tool was first calibrated using calibration marks present in the image and then used to measure the total thickness from the inner surface of the skin to the underlying muscle (total adipose thickness). Separate thickness measurements were made of each layer, identified as L1, L2 and L3. L1 is the outermost layer.

### Statistical methods

Statistical analysis was used to examine the effect of scan number on the contribution of L1, L2 and L3 to total adipose thickness. The ratio of each adipose layer to back fat thickness was determined for each pig at each scan. Let *y*_*ij *_denote the ratio of each adipose later to back fat thickness for the j'th (1, 2,...,9) pig at i'th (1,2,3) scan then the data was subjected to analysis of variance by the following linear mixed effects model:

*y*_*ij *_= *μ *+ *α*_*i *_+ *B*_*j *_+ *ε*_*ij*_

where

*μ *= overall mean

*α*_*i *_= effect of scan (1, 2 or 3)

*B*_*j *_= random effect of pig j 1, 2, 3,...9 ~ N(0, σB2
 MathType@MTEF@5@5@+=feaafiart1ev1aaatCvAUfKttLearuWrP9MDH5MBPbIqV92AaeXatLxBI9gBaebbnrfifHhDYfgasaacPC6xNi=xH8viVGI8Gi=hEeeu0xXdbba9frFj0xb9qqpG0dXdb9aspeI8k8fiI+fsY=rqGqVepae9pg0db9vqaiVgFr0xfr=xfr=xc9adbaqaaeGacaGaaiaabeqaaeqabiWaaaGcbaacciGae83Wdm3aa0baaSqaaiabdkeacbqaaiabikdaYaaaaaa@2FC9@)

*ε*_*ij *_= residuals ~ N(0, *σ*^2^)

When heteroscedastic errors were detected, the data was transformed (square root) and the statistical tests were done on the transformed data. The effect of scan was tested using the F test and multiple comparisons within scan were done by means of t-tests.

In addition the absolute thickness (cm) of each layer was related to body weight. Now, let *y*_*ij *_denote the thickness of the subcutaneous fat layer (L1, L2, L3, total) for the j'th pig at i'th scan. Further, let *x*_*ij *_denote the body-weight for the j'th pig at i'th scan and assume a linear relationship between y and x for each pig. Then the following random regression model is used to model the data:

*y*_*ij *_= *α *+ *A*_*j *_+ (*β *+ *B*_*j*_) * *x*_*ij *_+ *e*_*ij*_

[*A*_*j*_, *B*_*j*_]^*T *^~ N(0, Ψ)

*e*_*ij *_~ N(0, *σ*^2^)

Where *α *and *β *are, respectively, the fixed effects for the intercept and slope; [*A*_*j*_, *B*_*j*_]^*T *^are random effects vectors, assumed to be independent for different pigs; end *e*_*ij *_are independent identically distributed errors, assumed to be independent of the random effects.

The slope *β *is of biological interest because the parameter can be interpreted as the marginal effect of body weight on thickness of the subcutaneous fat layers i.e. Δ cm in the thickness of the fat layer per Δ Kg body weight.

This model was implemented in the statistical program "R", (Version 2.1.1) [9], together with the Non Linear Mixed Effects Models Package [10].

The effect of scan was tested using the F test and multiple comparisons within scan was done by means of t-tests.

## Results

**Figure 1:**
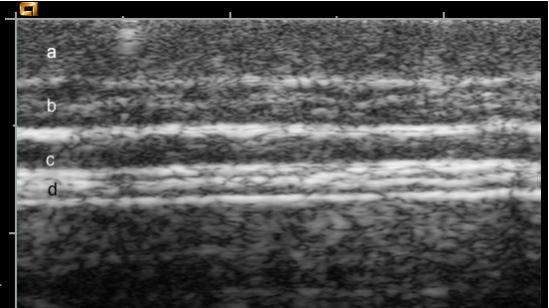
**Ultrasound image**. This image was obtained during the second scanning session. Layers are marked as follows. (a) skin, (b) outer, (c) middle and (d) inner, adipose layer.

Satisfactory ultrasonograms were obtained from all pigs in the study. A typical image is shown in Figure 1. The image shows that despite the excellent imaging capabilities of the machine used, differentiation between the edge of the outer aspect of L1 and skin is diffcult for the eye to identify. Differentiation here is based on the tissue structure, which for skin, is more uniform than for L1. The middle layer (L2) is composed of uniform hypoechoic tissue, producing few internal reflections. It has a sharp boundary with the overlying L1 and the underlying L3. Being hypoechoic (dark on the image) it contrasts well with the hyperechoic tissue of L1 and L3. This contrast with adjacent tissue and its sharp margins render L2 as well defined and easily recognized. L3 is readily identifiable. This layer contains a series of internal hyperechoic linear structures together with hypoechoic tissue. Being hyperechoic it contrasts well with L2 and also with the hypoechoic muscle fibers beneath. Its linear striations result in sharp edges, L3 is thus readily differentiated from adjacent tissues.

**Figure 2:**
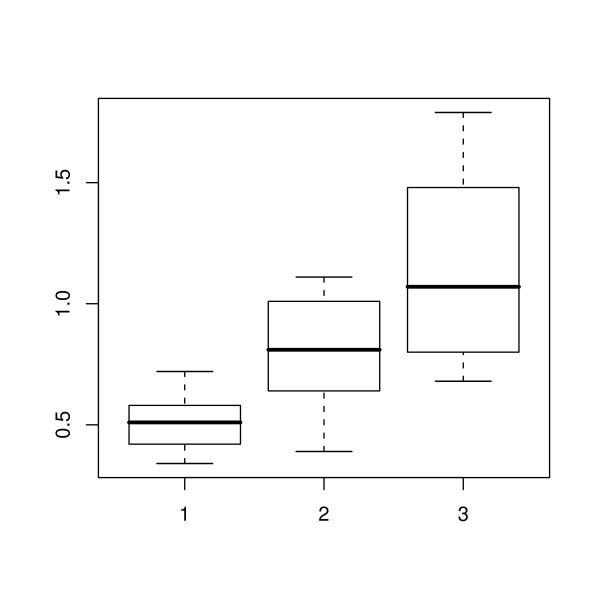
**Total adipose thickness**. Box-and-whisker plots for the total adipose tissue thickness (in cm) at the first, middle and last scanning session (1, 2 and 3 respectively), showing the 2.5, 25, 50, 75 and 97.5 % cumulative relative frequencies of the data.

**Figure 3:**
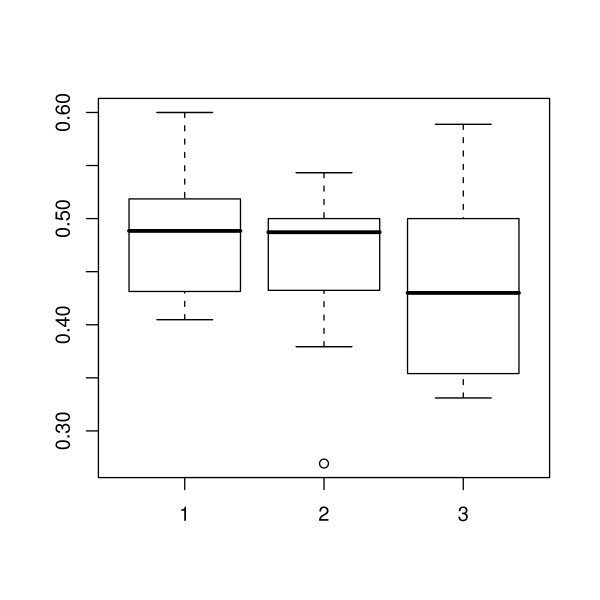
**Outer adipose layer thickness as a proportion of the total**. Box-and-whisker plots for the outer adipose layer showing the 2.5, 25, 50, 75 and 97.5% cumulative relative frequencies of the data. The plot shows the ratio of the outer adipose layer to the total adipose thickness at the first, middle and last scanning session (1, 2 and 3 respectively). Values outside the range of the whiskers are plotted individually.

**Figure 4:**
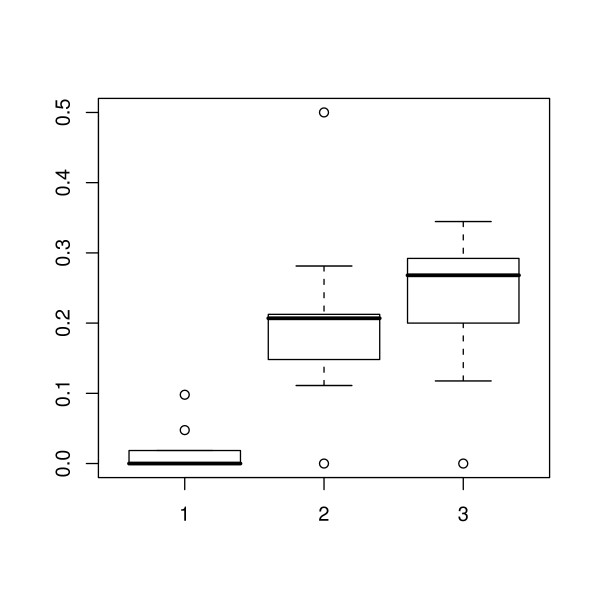
**Middle adipose layer thickness as a proportion of the total**. Box-and-whisker plots for the middle adipose layer showing the 2.5, 25, 50, 75 and 97.5% cumulative relative frequencies of the data. The plot shows the ratio of the middle adipose layer to the total adipose thickness at the first, middle and last scanning session (1, 2 and 3 respectively). Values outside the range of the whiskers are plotted individually.

**Figure 5:**
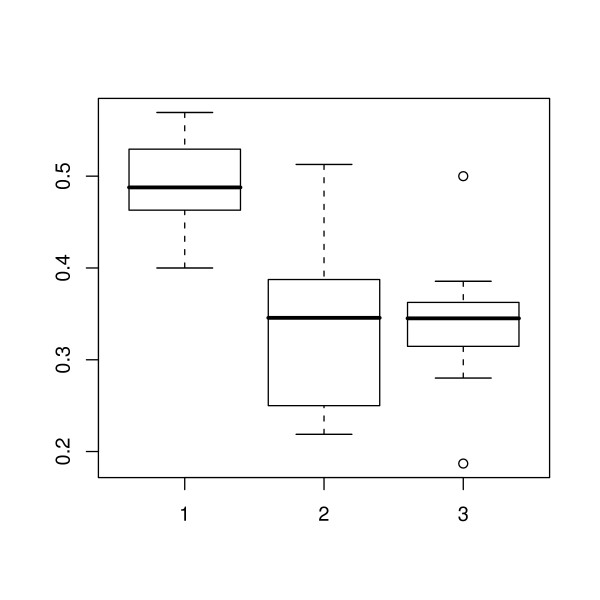
**Inner adipose layer thickness as a proportion of the total**. Box-and-whisker plots for the inner adipose layer showing the 2.5, 25, 50, 75 and 97.5% cumulative relative frequencies of the data. The plot shows the ratio of the inner adipose layer to the total adipose thickness at the first, middle and last scanning session (1, 2 and 3 respectively). Values outside the range of the whiskers are plotted individually.

During the period of the study there was an overall increase in total adipose thickness (Figure 2). The ratios of the thickness of each adipose layer to the total adipose thickness at the first, middle and final scan are shown in Figures 3, 4 and 5 respectively. The effect of scan on the square root of the ratio of the thickness of each layer to total adipose thickness was tested by the F test. The probability values for effect of scan were 0.397, < 0.0001 and < 0.0001, for L1, L2 and L3 respectively. Thus a statistically significant effect of scan was seen on L2 and L3. Comparisons of ratios between scans where significance was demonstrated are shown in Table 1. It can be seen that for both L2 and L3, significant changes were seen between the first scan and the two later scans, but not for either layer during the period between the final two scan sessions.

**Table 1:** Comparison of the contributions of the middle and inner adipose layers (L2 and L3, respectively) to the total adipose thickness at each scan time

Adipose layer	Scan	Estimate change	Lower CI (95%)	Upper CI (95%)	*P*
L2	1 vs 2	-0.07	-0.23	-0.004	0.014
	1 vs 3	-0.10	-0.27	-0.33	0.006
	2 vs 3	-0.002	-0.03	0.29	0.45
					
L3	1 vs 2	0.68	0.55	0.81	<0.001
	1 vs 3	0.70	0.76	0.84	<0.001
	2 vs 3	0.0002	-0.03	0.004	0.49

Test statistics are based on data from the linear regression model. "Estimate change" is the square of the alteration in the ratio of layer thickness to total adipose thickness between scans, estimated by the model. The later value is subtracted from the earlier value, so an increase in proportion is indicated by a minus sign. Ratios were transformed prior to fitting in the model; the statistical output for these ratios has been back transformed for this table."CI" indicates confidence interval." *P*" is the probability (with 12 degrees of freedom) that the difference between the transformed ratios at each scan is equal to zero.

The marginal effect of body weight on the thickness of each adipose layer and on total subcutaneous adipose tissue thickness, indicated by the estimated slope of the regression line together with its fit statistics is shown in Table 2. The change in thickness per unit change in body weight was greatest for L2, followed by L1 and L3 (0.0040, 0.0031, 0.0020 cm/Kg respectively).

**Table 2:** Relationship between the change in adipose thickness per unit change in body weight (cm/Kg) for each layer and for the total subcutaneous adipose thickness

Adipose layer	Slope	Lower CI (95%)	Upper CI (95%)	*P*
L1	0.0031	0.0024	0.0042	<0.001
L2	0.0040	0.0027	0.0057	<0.001
L3	0.0020	0.0009	0.0034	0.004

Total	0.0090	0.0065	0.0115	<0.001

Test statistics are based on data from the linear regression model. "Slope" can be interpreted as the marginal effect of body weight on thickness for each particular layer or for the total as indicated, i.e. the change in adipose thickness (in cm) per unit change in body weight in (Kg). Values for the confidence intervals (CI) and the probability (*P*) that the slop is not zero, are also given.

## Discussion

Meat quality is a function of the interplay between multiple variables and is of ongoing concern to pig producers, meat processors retailers and consumers alike [11]. Indicators of meat quality include pH, tenderness, intramuscular fat percentage and color. However the production of animals with high overall fat content is inefficient and is financially penalized as farmers are generally paid by weight after adjustments for the total body fat present are made. This has resulted in steps by the industry to optimize efficiency which include selecting for decreased backfat thickness. This in turn has lead to the production of meat with reduced palatability due to decreased fat content within the muscle [12].

While *in vivo *estimates of intramuscular fat content have been described [13], it has long been know that the depth of the innermost subcutaneous adipose layer is positively correlated with marbling scores in pigs [14]. Thus there is an interest in measuring the depth of particular adipose layers individually rather than all layers plus skin thickness as is current general practice.

Recent work [6] has identified a number of interesting features concerning the individual subcutaneous adipose layers in pigs. Heritability values for outer, middle and inner adipose tissue layers at the level of the 10th rib are 0.63, 0.45 and 0.53 respectively. The genetic correlations between these layers and the fat percentage of the longissimus dorsi muscle are small and probably not significantly different. Thus insofar as fat percentage is concerned, there may be little difference between selecting all layers or just individual layers, for genetic screening of breeding stock. The same authors [6] suggest that an emphasis during selection and during growth on the inner most adipose layer would both retain the usefulness associated with back fat measurements and be advantageous, since an increase thickness of the inner most layer is associated with marbling without an associated and wasteful increased is adipose tissue at other sites. Reports concerned with longevity of production sows have examined back fat thicknesses [1,15], the authors' however, are unaware of longevity studies that subdivide the back fat data into data for individual layers.

The results of this study indicate that for measurements made over time, the middle (L2) and inner (L3) adipose layers at the level of the last rib (P2 site) are more dynamic than the outer most layer (L1). As a proportion of the total, the outer layer was relatively static over the time period. This can be considered as "noise", from the point of view of measurement directed at monitoring change. The skin and the outer adipose layer do not contribute to proportional changes and its inclusion in measurements masks the magnitude of changes present in the deeper layers. Thus measurements that include either the inner or the inner plus middle layer are to be desired. This unequal rate of development of adipose layers is in agreement with that shown previously [16]. These authors showed by means of physical measurements made at serial slaughter procedure that back fat thickness varied with position on the animal and that the rate of growth of individual layers was non uniform.

When absolute rates of growth are considered (as opposed to contribution to total thickness), the middle layer was identified as being the most rapid growing of the three. This is shown in clearly in Table 2 which shows that the change in thickness per unit change in body weight is greatest in L2 followed by L1 and L3 respectively. Thus while L1 increased by greater amounts than L3 during the study, its contribution to total thickness changed less than was the case for either of the other two layers.

When body weight was included as a covariate in the analysis of variance model used to examine the effect of scan number on the contribution of L1, L2 and L3 to total adipose thickness, it was found to be non significant at all scan sessions. It was thus not included in the model shown here. We assumed in the statistical models that changes were linear over time, Table 1 suggests however that in this study the significant changes occurred early in the experiment. There may be a "time window" during which maximal changes occur. If this is so then there may also be an optimal time to effect changes to these inner layers by means of diet. More work is clearly indicated in this area.

Ultrasound technology is well established and has contributed much in the area of body composition in the swine industry [17]. Collection of data for individual adipose layers is more complex and time consuming than measurements of total backfat. The machine used in this study is a high level medical ultrasound machine. It is a large unit capable of extremely high spatial and contrast resolution but could not be considered a practical option for use under farm conditions. Many ultrasound machines, designed for use under such conditions are available either as amplitude (A) or brightness (B) mode units. They both allow measurement and the latter produces an image. Both methods often require multiple scanning attempts and a judgment by the operator as to the accuracy of the reading before it is possible to obtain data for individual layers. It is possible that improved algorithms will in future facilitate the measurement of individual layers to an extent where it becomes practicable.

## Conclusion

This study indicates that during growth, the middle and inner subcutaneous adipose layers change in the relative contribution they make to total back fat thickness and the middle layer shows the greatest increase in thickness per unit body weight. Ultrasound monitoring strategies would be better devoted to measurement of these individual layers than to the measurement of total back fat thickness.

## Competing interests

The author(s) declare that they have no competing interests.

## Authors' contributions

FM conceived and participated in the design of the study, carried out the ultrasound examinations and drafted the manuscript. AS performed the statistical analysis. MM participated in the design of the study and provided feeding and management protocols for the animals used. ES participated in the design of the study. All authors read and approved the final manuscript.
